# Editorial: Translational research in surgical applications and spinal tumors

**DOI:** 10.3389/fsurg.2024.1492713

**Published:** 2024-12-03

**Authors:** Madison J. Michles, Margot Martinez Moreno, Patricia L. Zadnik Sullivan, Ziya L. Gokaslan

**Affiliations:** Department of Neurosurgery, Rhode Island Hospital, Warren Alpert Medical School of Brown University, Providence, RI, United States

**Keywords:** spinal oncology, neurosurgical oncology, spinal tumor, translational research, chordoma

**Editorial on the Research Topic**
Translational research in surgical applications and spinal tumors

## Introduction

Advances in surgical techniques and equipment, oncological therapies, and genetic analyses have resulted in strides forward for the management of spine tumor patients (1, 2). The Research Topic “Translational Research in Surgical Applications and Spinal Tumors” provides an overview of diverse topics related to improving patient care and outcomes in spinal surgery and oncology. This editorial presents the key findings and takeaways from each article in this Research Topic, which aims to compile recent translational work on spinal tumors and their management.

## Quality of life and its selected determinants in the group of patients with surgically treated spinal tumors

The first article explores spine tumor patients' quality of life before and after spine stabilization and spinal cord decompression. This single-center prospective study found that patients' physical and psychological symptoms were significantly reduced after surgery and level of activity, disease acceptance, and perceived quality of life were significantly increased. This piece highlights the impacts of spine surgery on not only the physical but also emotional and psychological health of spinal oncology patients, which has important implications for patient recovery and satisfaction.

## An unusual presentation of ossified spinal meningioma: case report and literature review

The second article presents a rare case of ossified spinal meningioma, one of only 43 that have been cited until 2022. This case details the successful total resection of an ossified meningioma and highlights potential strategies for and obstacles to surgical resection as well as diagnostic mechanisms, all of which lead to improved management of these rare tumors.

## Clinical exploration of the international society of limb salvage classification of end-prosthetic failure using Henderson in the application of 3D-printed pelvic tumor prostheses

The third article compiles the results of a single-institution retrospective analysis of patients with 3D-printed pelvic tumor prostheses after tumor resection. The authors utilized the Henderson concepts for evaluating the efficacy of 3D-printed prosthetics and found that patients overall had satisfactory structural stability and effective function postoperatively. This paper demonstrates the potential for improved outcomes in pelvic tumor resection with the application of prosthetic devices designed based on the Henderson subtypes, which allows for both improved surgical construct planning and patient quality of life and function.

## Association of quantitative radiomic shape features with functional outcome after surgery for primary sporadic dorsal spinal meningiomas

The fourth article details a retrospective analysis of shape-based radiomic features of dorsal spinal meningiomas. The study found that increasing tumor sphericity was associated with improved neurological function following resection. These results suggest that preoperative shape-based analyses of spinal meningiomas could have both surgical planning and prognostic implications for patients, which could allow for better selection of surgical candidates and more accurate predictions of postoperative neurological status.

## Comparison of clinical efficacy of 3D-printed artificial vertebral body and conventional titanium mesh cage in spinal reconstruction after total en bloc spondylectomy for spinal tumors: a systematic review and meta analysis

The fifth article presents a meta-analysis comparing the efficacy of 3D-printed artificial vertebral bodies (AVBs) to conventional titanium mesh cages (TMCs) in spine tumor patients after total en bloc spondylectomy. The analysis revealed that surgeries utilizing 3D-printed AVBs had significantly improved intraoperative and postoperative outcomes than those performed with TMCs. These results suggest that the material used for reconstruction should be carefully considered by spine surgeons, as it could have a significant impact on postoperative function and patient satisfaction.

## Gross total resection and survival outcomes in elderly patients with spinal chordoma: a SEER-based analysis

The sixth article evaluates the survival outcomes in elderly patients with chordoma following gross total resection (GTR). This study found that although spine surgeons are often hesitant to perform aggressive surgical resections on elderly patients, patients 40–59 and 80–99 had a lower risk of mortality after GTR compared to patients who were managed nonoperatively. These findings support offering GTR to patients regardless of age, which can improve patient outcomes.

## Animal model considerations for chordoma research: reproducing the tumor microenvironment *in vivo* with humanized mice

The seventh article presents the current understanding of various humanized mouse models that are used in immunotherapy research for chordoma. Based on the literature, the authors suggest that humanized immunocompetent mice are better suited for studying the tumor microenvironment and subsequent immune involvement while immunocompromised mice are more useful for studying tumor growth and treatments independent of the immune system. This overview of various mouse models can be a helpful resource for researchers studying chordoma.

## Role of immunotherapy in treatment refractory chordomas: review of current evidence

The final article details the results of eight case studies and one clinical trial on the use of immunotherapy in treatment-refractory chordoma. The authors found that most patients in the case studies experienced temporary tumor regression and symptom improvement before the disease progressed, and the clinical trial observed little difference between the treatment and control arms such that it was discontinued. These results underscore the importance of continuing research into immunotherapies for chordoma as well as the need for increased understanding of the tumor microenvironment in order to design future studies.

## Conclusion

The articles compiled within this Research Topic illustrate a wide variety of advances, obstacles, and future directions for spinal surgery and oncology. While the exploration of genetic marker congruence between primary and metastatic spinal tumors remains an important area of study, this collection lacks an article dedicated to this complex issue, and the authors look forward to future works that can seek to shed light on this. Overall, this Research Topic not only highlights various exciting and contemporary topics in spine tumor treatment but also continues the conversation of translational research in spinal surgery and oncology to inspire the next generation of studies, techniques, and researchers.
